# Watching DNA Replication Inhibitors in Action: Exploiting Time-Lapse Microfluidic Microscopy as a Tool for Target-Drug Interaction Studies in *Mycobacterium*

**DOI:** 10.1128/AAC.00739-19

**Published:** 2019-09-23

**Authors:** Damian Trojanowski, Marta Kołodziej, Joanna Hołówka, Rolf Müller, Jolanta Zakrzewska-Czerwińska

**Affiliations:** a Faculty of Biotechnology, Department of Molecular Microbiology, University of Wroclaw, Wroclaw, Poland; b German Centre for Infection Research (DZIF), Hannover, Hannover-Braunschweig, Germany; c Helmholtz Institute for Pharmaceutical Research Saarland, Helmholtz Centre for Infection Research and Department of Pharmacy, Saarland University, Campus E8.1, Saarbrücken, Germany

**Keywords:** DNA replication inhibitors, *Mycobacterium*, antibiotics, bacterial chromosome, replisome

## Abstract

Spreading resistance to antibiotics and the emergence of multidrug-resistant strains have become frequent in many bacterial species, including mycobacteria, which are the causative agents of severe diseases and which have profound impacts on global health. Here, we used a system of microfluidics, fluorescence microscopy, and target-tagged fluorescent reporter strains of Mycobacterium smegmatis to perform real-time monitoring of replisome and chromosome dynamics following the addition of replication-altering drugs (novobiocin, nalidixic acid, and griselimycin) at the single-cell level. We found that novobiocin stalled replication forks and caused relaxation of the nucleoid and that nalidixic acid triggered rapid replisome collapse and compaction of the nucleoid, while griselimycin caused replisome instability, with the subsequent overinitiation of chromosome replication and overrelaxation of the nucleoid. In addition to study target-drug interactions, our system also enabled us to observe how the tested antibiotics affected the physiology of mycobacterial cells (i.e., growth, chromosome segregation, etc.).

## Abstract (precis)

Spreading resistance to antibiotics and the emergence of multidrug-resistant strains have become frequent in many bacterial species, including mycobacteria, which are the causative agents of severe diseases and which have profound impacts on global health.

## INTRODUCTION

Bacterial resistance to antibiotics, which is an increasing health problem worldwide, is a concern for all commercially used antimicrobials (1–7). Recent years have seen the constant emergence of new strains that are resistant to multiple drugs, including last-resort antibiotics (8). Moreover, only a few new antimicrobial drugs have been approved in the past 10 years. Therefore, novel antibiotics are urgently needed.

Antibiotics target basic cellular processes, such as the synthesis and integrity of the cell wall (penicillins, cephalosporins, lipoglycopeptides, polymyxins, etc.), transcription (rifampin), translation (aminoglycosides, macrolides, lincosamides, tetracyclines, oxazolidinones), and metabolic pathways (sulfonamides, diaminopyrimidines) (9–13). Since the proteins that govern bacterial DNA replication differ from their eukaryotic counterparts, chromosome replication represents another promising therapeutic target (14–17). However, bacterial chromosome replication is targeted by only a few current antibiotics, such as quinolones, aminocoumarins, and metronidazole (18–23). Recently, it has also been reported that nonsteroidal anti-inflammatory drugs exert an inhibitory effect on bacterial chromosome replication (24).

The genus *Mycobacterium* encompasses both human and animal pathogens (Mycobacterium leprae, M. tuberculosis, M. bovis) that cause severe diseases and that have profound impacts on global health and the world economy. Although the number of new M. tuberculosis infection cases has been decreasing annually (25), tuberculosis (TB) remains one of the most prominent causes of death worldwide and the main cause of death among HIV-infected individuals (26). It is estimated that one-third of the human population is latently infected with M. tuberculosis and that tubercle bacilli may be reactivated from the latent state upon immunosuppression later in life (27). As with other pathogens, the resistance of M. tuberculosis is becoming a serious obstacle in effective drug therapy. According to the WHO, of the 10 million new TB cases in 2016, nearly half a million were classified as multidrug resistant (MDR; resistant to at least two anti-TB drugs), and among those cases, about 6% were caused by extensively drug-resistant (XDR) strains (strains resistant to more than four anti-TB medications) (26). The increasing number of resistant M. tuberculosis strains coupled with the short list of anti-TB drugs prompted researchers to reevaluate some commonly used antibiotics (e.g., linezolid, clofazimine, amoxicillin-clavulanate) for off-label treatment of TB (28, 29). The serious challenge in the treatment of TB arises from the distinctive mycobacterial cell biology (30–38). Asymmetric growth (mycobacteria elongate preferentially from the old pole) and septum placement give rise to daughter cells of unequal sizes and growth rates and also different susceptibilities to antibiotics (30, 39, 40). Thus, it is important for researchers to explore how anti-TB drugs act on individual cells.

**FIG 1:**
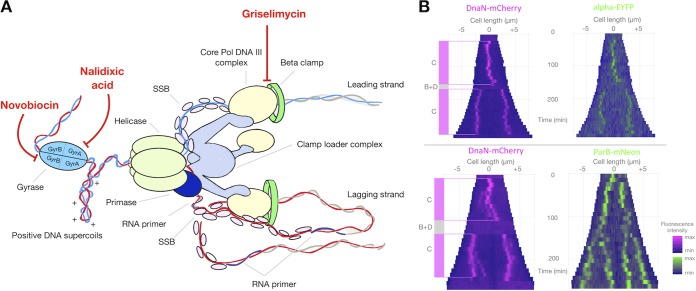
Replisomes are highly dynamic entities in M. smegmatis. (A) Schematic representation of the replisome, which is a multiprotein complex engaged in DNA replication. Core DNA polymerase III (core DNA Pol III) is loaded by the clamp loader complex via the tau subunit, whereas the beta clamp is loaded via the delta subunit of the clamp loader complex. The catalytic subunit alpha of core DNA Pol III interacts with the beta clamp in its hydrophobic cleft to give the replisome a high degree of processivity. Both Ndx and novobiocin target DNA gyrase. Novobiocin binds to the GyrB subunit, while Ndx binds to the gyrase/DNA complex (the cleavable complex). Griselimycin abolishes the alpha-beta interactions by binding in the hydrophobic cleft of the sliding clamp. (B) Kymographs of representative cells from two M. smegmatis strains: DnaN-mCherry/Alpha-EYFP (top) and DnaN-mCherry/ParB-mNeon (bottom). The fluorescence intensities over time are depicted in magenta for DnaN-mCherry and in green for Alpha-EYFP or ParB-mNeon. “C period” refers to the time during which DnaN-mCherry and/or Alpha-EYFP is observed as diffraction-limited foci, while “B+D period” refers to the time between the termination of DNA replication and the initiation of another round of replication in the next generation.

Here, we present a system that combines time-lapse microfluidic microscopy (TLMM) and replisome-tagged (Fig. 1A) fluorescent strains of M. smegmatis to allow the real-time observation of how antibiotics affect chromosome replication at a single-cell level. We show how the replisome and chromosome dynamics are altered upon the addition of novobiocin (an aminocoumarin), nalidixic acid (Ndx; a quinolone), and griselimycin (GM; a novel antimicrobial agent) (41), all of which belong to the DNA replication inhibitor class. The system described herein allows researchers to simultaneously observe the target along with other processes (e.g., cell growth and chromosome segregation) and thus provides additional results beyond the simple measurement of target protein inhibition. It may also give mechanistic insights into the mode of action of the studied drugs.

## RESULTS

### TLMM allows chromosome and replisome dynamics to be observed in real time during antibiotic treatment.

In this study, we used fluorescent reporter strains of M. smegmatis and a microfluidic CellASIC Onix platform to observe in real time the actions of novobiocin, nalidixic acid, and griselimycin at the single-cell level. The bacterial replisome (a multiprotein complex that is involved in chromosome replication) and the targets of the studied antibiotics are schematically depicted in Fig. 1A. Both nalidixic acid (Ndx) and novobiocin affect replisome passage indirectly by inhibiting the enzymatic activity of DNA gyrase, which normally triggers the relaxation of positive supercoils ahead of the replication fork to resolve the torsional tension and to allow DNA synthesis to proceed. Although the two drugs act on the same target, Ndx prevents the religation of cleaved DNA by binding to the gyrase/DNA cleavable complex, resulting in double-strand breaks (42, 43), whereas novobiocin competes with ATP for binding to the GyrB subunit and thus inhibits cleavage but not the binding of DNA (44–46). In contrast to novobiocin and Ndx, griselimycin acts directly on the replication machinery; it prevents the interaction between the beta clamp and the catalytic subunit alpha of DNA polymerase III (Pol III), altering the processivity of the replisome (41). We used previously constructed strains in which replisome subunits (the beta clamp and/or catalytic subunit alpha), a chromosomal marker (HupB), and/or *oriC* (ParB bound to *oriC*-proximal *parS* sites) was tagged with different fluorescent proteins (FPs) (47–50). The strains used in this study are presented in Table S1 in the supplemental material. The growth of all strains was similar to that of the wild-type (WT) M. smegmatis mc^2^155 strain (Fig. S1, bottom right). We calculated the inhibitory concentration (IC) of the three tested antibiotics for all fluorescent reporter strains using a Bioscreen C instrument (see Materials and Methods and Fig. S2). The concentrations at which growth was inhibited by 50% (IC_50_s) are presented in Table 1. For novobiocin and griselimycin, the IC_50_ values were lower for the reporter strains than for WT mc^2^155 cells, indicating that the reporter strains had higher susceptibilities to these antibiotics.

**TABLE 1:** IC_50_s of tested antibiotics for the various strains used in the study

Strain	IC_50_ (μg/ml)		
	Novobiocin	Nalidixic acid	Griselimycin
mc^2^155	4.8	50	0.43
DnaN-mCherry/ParB-mNeon	4.0	50	0.22
DnaN-mCherry/Alpha-EYFP	1.2	50	0.12
DnaN-mCherry/HupB-EGFP	2.9	49	0.17

In our experiments, we used concentrations of 5× and 10× IC_50_, which were expected to trigger discrete and observable changes without rapidly killing the mycobacterial cells. Cells loaded into microfluidic chambers were observed using the same protocol: 5 h of growth under optimal conditions followed by 5 h of antibiotic treatment (this constituted approximately twice the chromosome replication time) and 7 h of washout. In the absence of any antibiotic, cells usually initiated one replication round per cell cycle (excluding the 10 to 15% multifork cells, in which another round of replication is initiated before the previous round has finished, which was consistent with the findings of previous studies [47, 48]). Replication initiation corresponded to the appearance of a fluorescent spot that was positioned slightly asymmetrically to the middle of the cell. Under our experimental conditions, replication lasted for 119 ± 16 min (mean ± standard deviation [SD], *n* = 60) in the DnaN-mCherry/ParB-mNeon strain and 149 ± 9 min (*n* = 64) in the DnaN-mCherry/Alpha-EYFP strain (C period). During the C period, we observed that replisomes frequently split and merged back together. Thereafter, replication was terminated, which was observed as the disappearance of the DnaN-mCherry and/or Alpha enhanced yellow fluorescent protein (Alpha-EYFP) signal. This was followed by a period during which the fluorescence signal of the tagged replisomes was dispersed (B+D period); it lasted 27 ± 10 min (*n* = 60) in the DnaN-mCherry/ParB-mNeon strain and 13 ± 11 (*n* = 81) min in DnaN-mCherry/Alpha-EYFP strain (see the kymographs in Fig. 1B).

### Novobiocin stalls replication forks but only moderately affects the cell elongation rate.

**FIG 2:**
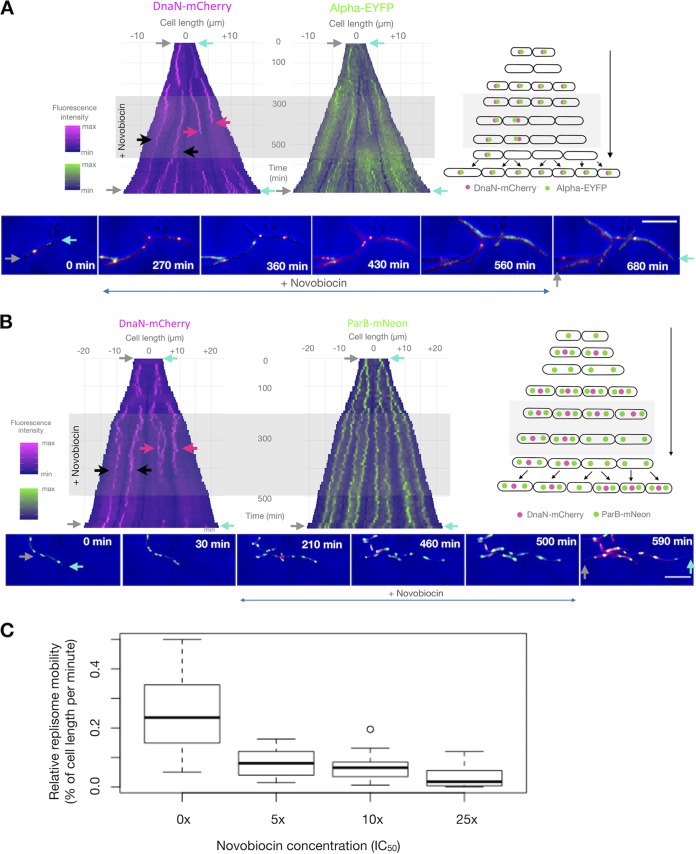
Novobiocin stalls replisomes but has only a moderate effect on growth in M. smegmatis cells. (A and B) Kymographs of representative cells of two M. smegmatis strains exposed to novobiocin: DnaN-mCherry/Alpha-EYFP (A) and DnaN-mCherry/ParB-mNeon (B). The left kymographs show the DnaN-mCherry fluorescence over time (A and B), while the right ones present the Alpha-EYFP (A) or ParB-mNeon (B) fluorescence over time. Time-lapse images of the cells depicted on the kymographs are presented at the bottom, and schematic graphs of the analyzed cells are presented on the right. Antibiotic treatment is indicated by gray rectangles; black arrows mark cells in which replication proceeds in the presence of novobiocin; red arrows mark cells in which replication terminates during novobiocin treatment; gray and cyan arrows indicate the corresponding poles on both images and kymographs. Bars, 5 μm. (C) Dose-dependent inhibition of replisome mobility during novobiocin exposure of DnaN-mCherry/ParB-mNeon cells. The box plots represent replisome mobility (defined as the relative distance that a replisome traveled along the cell per minute) under increasing concentrations of novobiocin (0×, 5×, 10×, and 25× IC_50_; *n* = 20 for each concentration). Measurements were performed from the old pole.

In the presence of novobiocin, the replisomes (both Alpha-EYFP and DnaN-mCherry) remained visible but exhibited significantly decreased mobility along the cell (Fig. 2A to C; Movie S1). Because Alpha-EYFP fusion causes prolongation of the C period and shortening of the B+D period (48) (see the kymographs in Fig. 1B), we used DnaN-mCherry/ParB-mNeon to analyze the changes in replisome dynamics during novobiocin treatment. After the addition of novobiocin, the mobility of the replisomes decreased in a dose-dependent fashion (Fig. 2C). At a dose of 25× IC_50_ of novobiocin, the replisomes were almost completely stalled. As a consequence of replication fork slowing, the C period (in cells that had terminated replication during novobiocin treatment but not in untreated cells) was profoundly prolonged at both the 5× IC_50_ and the 10× IC_50_ of novobiocin (mean, 175% ± 60% [*n* = 47] and 192 ± 57% [*n* = 67], respectively). Two major groups of cells were observed during novobiocin exposure, as depicted in the representative kymographs presented in Fig. 2A and B. The first group (45% of 92 cells in the 5× IC_50_ group and 34% of 187 cells in the 10× IC_50_ group) comprised cells in which replisome foci were visible throughout the antibiotic treatment due to the delay in replication fork passage (black arrows on the kymographs in Fig. 2A and B). The second group comprised cells that terminated replication in the presence of novobiocin (55% and 66% at 5× IC_50_ and 10× IC_50_, respectively; Fig. 2A and B, red arrows). In the latter group, 81% of the cells in the 5× IC_50_ group (*n* = 40) but only 17% of the cells in the 10× IC_50_ group (*n* = 20) initiated the next replication round during antibiotic treatment. It is important here to discriminate between the termination and the subsequent initiation of a new replication round and between replication restart after fork collapse. Since a new round of replication started near the midcell region of two daughter cells almost simultaneously, the distance between two replisomes corresponding to new replication rounds was far longer than that between split replisome spots engaged in the same replication round. Therefore, we were able to exclude the replication restart after replisome collapse. In cells terminating replication and starting a new replication round under novobiocin treatment, we observed significant prolongation of the B+D period (86 ± 74 min and 101 ± 64 min at 5× IC_50_ and 10× IC_50_, respectively; *P* < 0.005 for both groups, which was true by a pairwise comparison *t* test with the pooled SD and a pairwise comparison using the Wilcoxon rank-sum test). In those cells, we clearly observed splitting of the ParB-mCherry foci, suggesting that the segregation of nascent *oriC* regions was not significantly impaired (see the kymograph in Fig. 2B). During novobiocin exposure, cells elongated only ∼30% (1.07 ± 0.38 μm/h in the 5× IC_50_ group, *n* = 49) to ∼50% (0.70 ± 0.44 μm/h in the 10× IC_50_ group, *n* = 48) more slowly than they did under optimal conditions (1.50 ± 0.38 μm/h, *n* = 50). Interestingly, in 12% of cells (*n* = 177), during the washout period we observed additional spots of DnaN-mCherry that did not colocalize with Alpha-EYFP, indicating that the beta clamp may be involved in processes other than chromosome replication (e.g., DNA repair) and/or that residual sliding clamps accumulate on the lagging DNA strand, as shown previously (51–54).

### Addition of nalidixic acid results in growth arrest and replisome disassembly.

**FIG 3:**
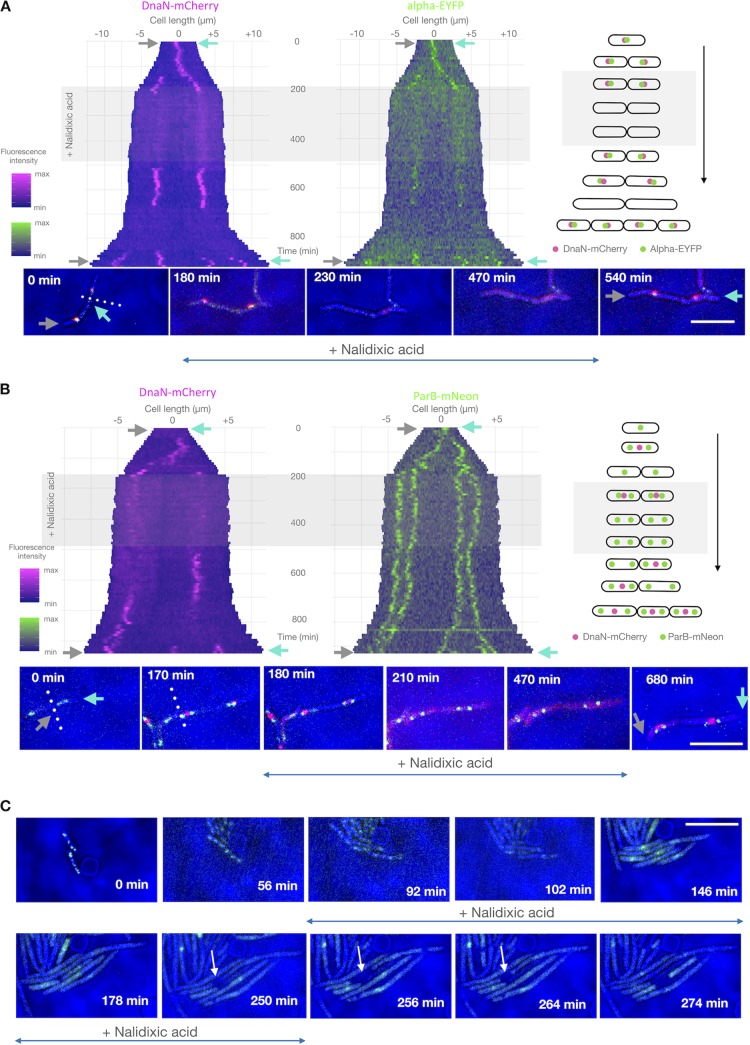
Nalidixic acid treatment results in replisome disassembly and growth arrest of M. smegmatis cells. (A and B) Kymographs of representative cells of two M. smegmatis strains exposed to Ndx: DnaN-mCherry/Alpha-EYFP (A) and DnaN-mCherry/ParB-mNeon (B). The left kymographs show the DnaN-mCherry fluorescence over time, while the right ones present the Alpha-EYFP (A) or ParB-mNeon (B) fluorescence over time. Time-lapse images of the cells depicted on the kymographs are presented at the bottom, and schematic graphs of the analyzed cells are presented on the right. Antibiotic treatment is indicated by the gray rectangle, gray and cyan arrows indicate the corresponding poles on both images and kymographs, and the dotted line indicates the boundary between two daughter cells. (C) TLMM analysis of E. coli cells harboring the YPet-DnaN fusion, as assessed during Ndx treatment. Bars, 5 μm.

In contrast to novobiocin, the addition of Ndx (at both 10× IC_50_ and 5× IC_50_) resulted in the rapid loss of foci (both Alpha-EYFP and DnaN-mCherry; Fig. 3A and B; Movie S2). The loss of foci most likely reflects the collapse of replisomes. The timing of replisome spot(s) disappearance was dose dependent, occurring at 40 min at 10× IC_50_ but 214 min at 5× IC_50_ (*n* = 45 and 50 cells, respectively). As a consequence of replication fork collapse, the *oriC* regions failed to properly segregate. This is intuitive, as after replisome disassembly newly replicated regions do not emerge behind the forks, and that stops the segregation process. As shown in Fig. 3B, in cells that had begun replicating shortly before being switched to Ndx-containing medium, the ParB complexes remained in close proximity rather than reaching their native positions proximal to the cell poles. This pole-proximal localization of ParB-mNeon foci was not restored in these cells until very late during the washout period. After Ndx washout, replisomes appeared at the same site from which they had previously disappeared in approximately 70% of cells in both the DnaN-mCherry/ParB-mNeon and DnaN-mCherry/Alpha-EYFP strains (*n* = 100 per strain). This confirms our conclusion that the addition of Ndx led to replication fork collapse and that replication was restarted at the same site after Ndx removal.

M. smegmatis cells stopped growing soon after the addition of Ndx, and the timing of growth restoration during the washout period was not dependent on the concentration of the drug utilized. This growth arrest in M. smegmatis was strikingly different from our results obtained in Escherichia coli (with the YPet-DnaN strain, kindly provided by Rodrigo Reyes-Lamothe [55]) subjected to a similar Ndx treatment scheme (the exception was the use of a 2-h Ndx exposure, which was also twice the duration of the C period in this bacterium [56]). Consistent with previous reports (57, 58), we observed that E. coli cells became filamentous during Ndx treatment, indicating that there was dysregulation between cell growth and division. Additionally, in our study, a single YPet-DnaN focus appeared transiently in the central part of each filamentous E. coli cell (Fig. 3C). It remains unclear whether the YPet-DnaN foci observed in Ndx-treated E. coli cells are due to the replication holdup rather than the involvement of the beta clamp in the DNA repair of double-strand breaks. Interestingly, the elongating Ndx-exposed E. coli cells observed in the differential interference contrast (DIC) channel exhibited the transient formation of areas that presumably reflected differences in the density of the cytoplasm (marked with an arrow in Fig. 3C). This was not accompanied by any disruption of cell integrity, as all cells were viable and their growth was not arrested. Similar observations were also described in previous studies (57), in which the above-mentioned changes were regarded as vacuole-like structures.

### Griselimycin affects replisome processivity and leads to the formation of *oriC*-proximal loops.

**FIG 4:**
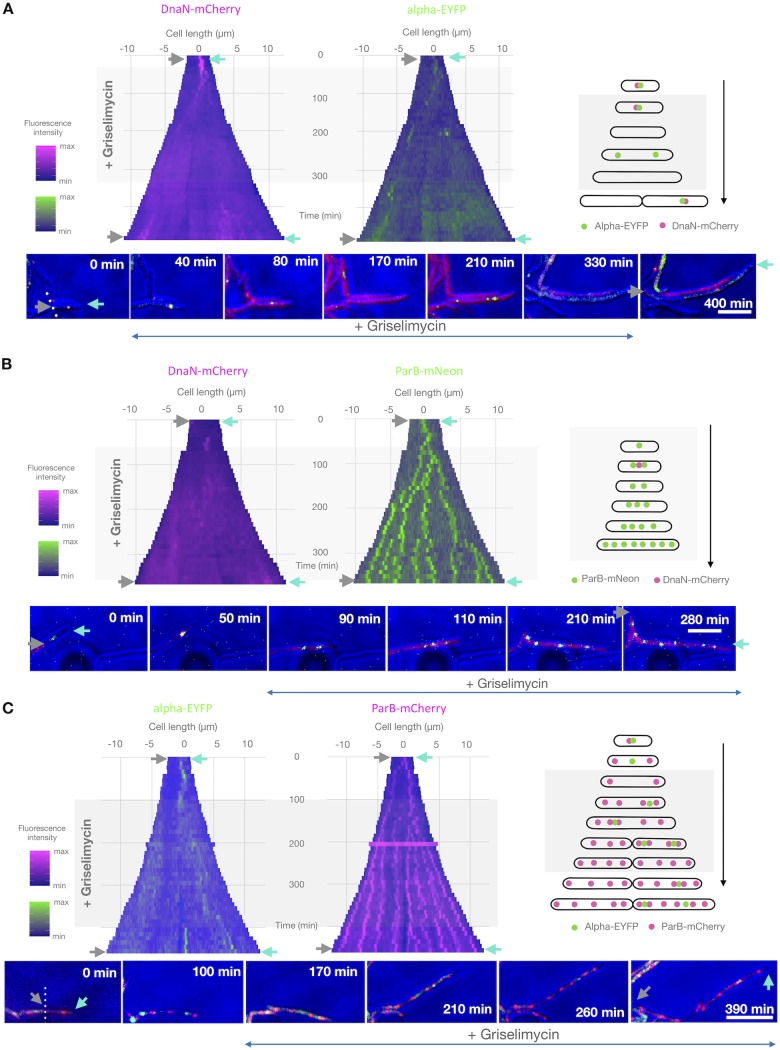
Griselimycin treatment results in the loss of replisome processivity and multiplication of *oriC*-proximal regions. Kymographs of representative cells of three M. smegmatis strains exposed to GM: DnaN-mCherry/Alpha-EYFP (A), DnaN-mCherry/ParB-mNeon (B), and Alpha-EYFP/ParB-mCherry (C). The left kymographs show the fluorescence of DnaN-mCherry (A, B) or Alpha-EYFP (C) over time, while the right ones present the fluorescence of Alpha-EYFP (A), ParB-mNeon (B), or ParB-mCherry (C) over time. Time-lapse images of the cells depicted on the kymographs are presented at the bottom, and schematic graphs of the analyzed cells is presented on the right. Antibiotic treatment is indicated by the gray rectangle, gray and cyan arrows indicate the corresponding poles on both images and kymographs, and a dotted line indicates the boundary between two daughter cells. Bars, 5 μm.

As mentioned above, griselimycin (GM) potently inhibits the interaction of the beta clamp with the catalytic subunit alpha in the core of DNA Pol III (Fig. 1A). Thus, the DnaN-mCherry/Alpha-EYFP strain was ideal for investigating the action of GM, as the interacting proteins are tagged with different fluorophores in this strain. Based on a previous report (41), we expected GM to block the proper assembly of replisomes *in vivo*. Indeed, after GM was added to M. smegmatis cells, the DnaN-mCherry foci rapidly disappeared and the fluorescence signal remained diffuse for the rest of the antibiotic treatment period in all analyzed cells (*n* = 50) (Fig. 4A to C; Movie S3). Strikingly, we also observed several appearances of short-lasting Alpha-EYFP fluorescent foci during GM exposure; the lifetimes of these foci were much shorter than the C period in the absence of antibiotic treatment, suggesting that cells presenting such foci underwent abortive replication or replication restart after replisome collapse. To investigate this further, we used an additional reporter strain that expressed Alpha-EYFP and ParB-mCherry from its native chromosomal loci. TLMM analysis revealed that during GM treatment, the appearance of the Alpha-EYFP foci was accompanied by ParB-mCherry (i.e., *oriC*) duplication, suggesting that a new round of replication had been initiated. The Alpha-EYFP foci disappeared soon after duplication of the ParB-mCherry complex (average lifetime of the Alpha-EYFP foci, 39 ± 20 min in the 10× IC_50_ group, as assessed at 2-min frame intervals; *n* = 47). During GM treatment, we observed up to several duplication events at the *oriC* region, each of which was preceded by the colocalization of Alpha-EYFP with ParB-mCherry. To emphasize, we did not observe the colocalization of DnaN-mCherry with Alpha-EYFP foci in DnaN-mCherry/Alpha-EYFP cells exposed to GM. Hence, during GM exposure, the incomplete replisome can assemble at the *oriC* region, but its inability to interact with the sliding clamp results in the loss of processivity of the core polymerase and consequent abortive replication. We assume that several loops containing an *oriC*-proximal region might therefore arise (which is consistent with the presence of multiple ParB-mCherry foci in a single cell; Fig. 4C), and the length of these loops may correlate with the lifetime of individual Alpha-EYFP foci.

### Chromosome dynamics is differentially affected by the various replication inhibitors.

**FIG 5:**
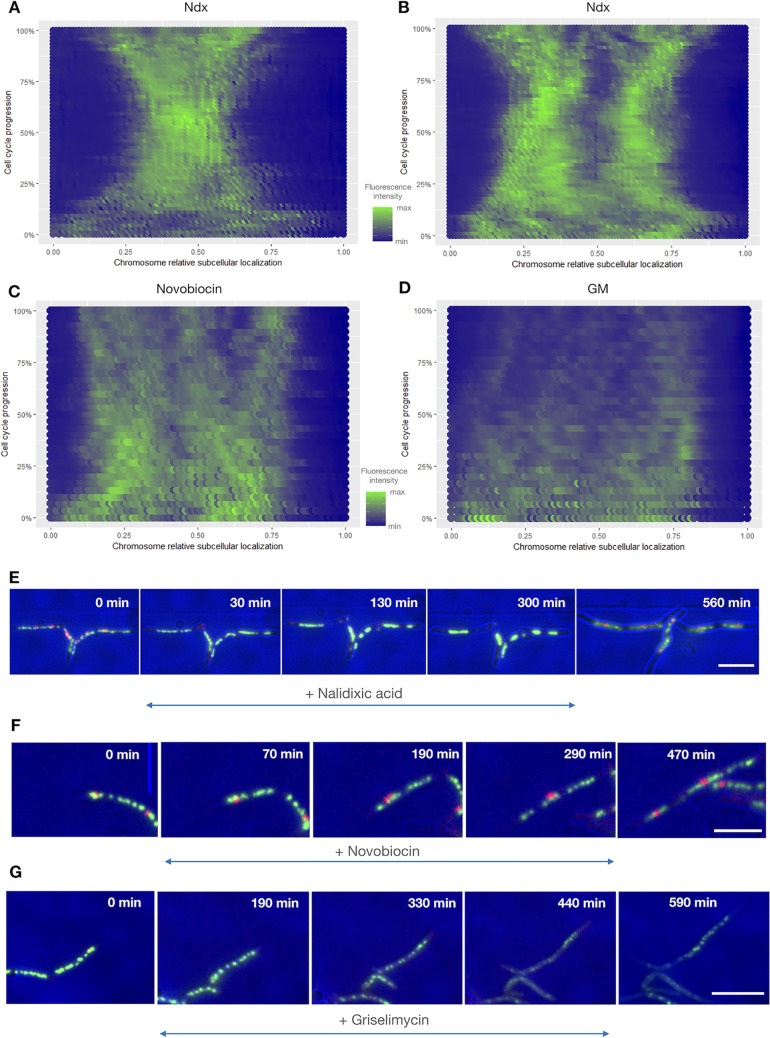
All three analyzed antibiotics affect chromosome organization and dynamics. (A to D) Fluorescence profiles of HupB-EGFP over time for representative cells (*n* = 5) under treatment with the different antibiotics. (A) In cells with an overall replication progression of <50% (cells in which replication progressed beyond the halfway point), Ndx exposure causes the chromosome to shrink until only a single fluorescent cluster is visible. (B) In cells with an overall replication progression of >50%, Ndx exposure causes the chromosome to shrink until two fluorescent clusters are visible. (C) During novobiocin treatment, chromosome decondensation is visualized as separate fluorescent clusters. (D) During GM treatment, chromosome decondensation is followed by a decrease in the fluorescence intensity. (E to G) TLMM images of representative cells under treatment with Ndx (E), novobiocin (F), or GM (G). Bars, 5 μm.

In addition to its effects on transcription, DNA replication influences the overall nucleoid organization (59–63). We thus examined how antibiotic-triggered replisome collapse (Ndx), replication fork slowdown (novobiocin), or the loss of processivity (GM) affected the nucleoid structure. To answer these questions, we used the DnaN-mCherry/HupB-EGFP strain, which allowed us to simultaneously observe chromosome dynamics (HupB is a homolog of the HU protein from E. coli, which occupies the whole nucleoid and is used as a chromosomal marker [49, 50, 64]) and track the progression of replication. Our previous studies demonstrated that HupB tagged with enhanced green fluorescent protein (EGFP) (HupB-EGFP) does not alter the mycobacterial chromosome structure and that the nucleoid adopts a bead-like pattern spread along the cell (49). The positioning of the nucleoid is asymmetric, being closer to the new cell pole for almost the entire cell cycle (50). Antibiotic exposure of DnaN-mCherry/HupB-EGFP cells yielded replication patterns similar to those described in the other strains of M. smegmatis exposed to each drug (see above). Interestingly, we observed that the various antibiotic-induced alterations in replisome dynamics reflected different changes in chromosome organization (Fig. 5 and Movie S4).

The area occupied by the mycobacterial chromosome, which was measured by comparing the total area occupied by HupB-EGFP before and during antibiotic treatment, was decreased by exposure to nalidixic acid (10× IC_50_) (Fig. 5A, B, and E). The chromosome shrank rapidly after Ndx was introduced into the medium (29 ± 7.5 min, *n* = 57) and then decondensed slowly during washout, beginning at 120 min after the removal of Ndx. The chromosome was condensed to approximately 30% of its initial size (27% ± 20%, *n* = 49), which accounts for the observed increase in the fluorescence intensity of HupB-EGFP. Interestingly, in cells that initiated replication less than 60 min before being switched to Ndx-containing medium (i.e., those in which replication had progressed less than halfway), the chromosome area shrank to the point that it was visualized as a single fluorescent cluster per cell (Fig. 5A and the left cell in Fig. 5E). In contrast, in the cells in which replication had proceeded more than halfway before the addition of the drug (i.e., those that initiated replication a minimum of 60 min before Ndx addition), the chromosome was compacted to the point that we observed two separate fluorescent clusters (Fig. 5B and the right cell in Fig. 5E). These clusters presumably reflected newly replicated sister chromosome regions. In this, our observations parallel the previous description of bilobed chromosomes in slowly growing E. coli cells (65, 66). The characteristic bead-like structure of the mycobacterial chromosome was lost upon Ndx treatment and was replaced by a uniform fluorescent patch(es). Chromosome compaction and replisome disassembly together explain why the nascent *oriC* regions remained in close proximity throughout Ndx treatment and even longer after its removal. The changes in nucleoid density were reversible in all observed cells, and the chromosome regained its normal morphology (i.e., a bead-like pattern) after Ndx washout. Our observations are in line with those previously obtained in Ndx-exposed E. coli cells (57, 58), which showed that the chromosome was compacted around the midcell. Compaction of the nucleoid was also reminiscent of the changes seen in E. coli cells exposed to UV light (67). However, we did not observe decondensation of the chromosome during prolonged Ndx exposure, as was previously reported in E. coli (57). We hypothesize that the decondensation seen in Ndx-treated E. coli might reflect fragmentation subsequent to the introduction of double-strand breaks.

In contrast to the effects observed in Ndx-treated cells, those exposed to novobiocin (*n* = 100) exhibited preservation of the bead-like chromosome structure throughout the antibiotic treatment (Fig. 5C and F). However, we observed chromosome decondensation, as the area occupied by the nucleoid was extended. The chromosomes of novobiocin-treated cells formed clusters consisting of regular HupB-EGFP foci, which were further apart along the long cell axis than the foci of actively replicating cells growing in novobiocin-free medium. Decondensation was presumably a result of increased cell volume (cells were still elongating in the presence of novobiocin). Notably, the intensities of the HupB-EGFP foci were not significantly decreased. This may indicate that newly replicated sister chromosomes are more likely to separate into longer cells.

In cells exposed to GM (*n* = 155), the chromosome structure underwent dynamic rearrangements. As seen for novobiocin treatment, GM treatment triggered chromosome decondensation; however, the area occupied by the nucleoid was much larger under GM exposure than under novobiocin exposure (Fig. 5D and G). In most GM-treated cells, the HupB-EGFP foci were heterogeneous in size and intensity, and they were distributed unevenly along the cell (Fig. 5G, top cell). Moreover, in 17% of the cells (*n* = 155), the bead-like pattern of the HupB-EGFP complexes was lost and only dispersed fluorescence was observed (Fig. 5G, bottom cell). This might reflect alteration in HupB binding to the chromosome and/or overrelaxation of the chromosome, which filled more space in growing filamentous cells. To test the first possibility, we treated cells with GM for 5 h and performed Hoechst 33342 staining. The staining overlapped the fluorescence arising from the HupB-EGFP complexes (Fig. S3), indicating that there was no change in the binding of HupB to the chromosome of GM-treated M. smegmatis cells. This suggests that the dispersed HupB-EGFP fluorescence seen in some cells likely reflects a change in chromosome organization. We speculate that such profound changes may, at least partially, have arisen from the induction of DNA damage, as seen in Ndx-treated E. coli strains (57). Indeed, further analyses revealed that 50% of cells that exhibited dispersed fluorescence (*n* = 20) did not elongate further; notably, their dispersed fluorescence signal was preceded by the sudden condensation of the nucleoid to a single bright HupB-EGFP spot, which then rapidly disappeared. The remaining 50% of cells continued to grow, and 37% of them regained the normal chromosome structure after GM washout.

## DISCUSSION

In this work, using time-lapse microfluidic microscopy (TLMM) and a set of fluorescent reporter strains, we show how drugs affecting replication alter the replisome and chromosome dynamics of mycobacterial cells. Single-cell studies are needed to shed new light on bacterial physiology during antibiotic exposure and may broaden our understanding of how bacterial cells escape killing by antibiotics and/or how tolerance/resistance to antimicrobial drugs may develop. Our present work reveals that combining single-cell techniques with appropriate target-tagged strains can provide new insights into the action of well-known compounds (novobiocin, nalidixic acid), as well as novel compounds (griselimycin). We chose M. smegmatis as a model for studying the biology of the tubercle bacilli, some of which (e.g., M. tuberculosis, M. bovis) cause severe diseases. The use of such a model is important because the mycobacterial mode of growth substantially differs from the corresponding modes of growth of extensively studied model bacteria, e.g., E. coli, Bacillus subtilis, and Caulobacter crescentus.

We tested three drugs affecting replication (Fig. 1), nalidixic acid (Ndx), novobiocin, and griselimycin (GM), all of which had various impacts on the dynamics of the replication complex. Novobiocin slowed the replisomes (observed as a prolongation of the C period and a decrease in the subcellular mobility of the tagged replisomes) and moderately decreased cell elongation. In contrast, Ndx completely abolished replication, as indicated by the disappearance of the replisome foci. Surprisingly, unlike the situation in E. coli cells (Fig. 3C) (57, 58), Ndx caused the growth arrest of mycobacterial cells. This may suggest that the changes induced by Ndx may differ between bacteria.

Finally, we found that GM aborted the interaction of the core DNA polymerase III complex with the sliding clamp, which was observed as a loss of colocalization for DnaN-mCherry and Alpha-EYFP. The loss of DnaN-mCherry foci is remarkable, as a beta clamp needs to be disassembled by the clamp loader and one would expect to observe the presence of DnaN foci during GM treatment, an action that corresponds to the binding to hydrophobic clefts present on the DnaN ring (41). These clefts contribute to both catalytic subunit interactions and the binding of the clamp loader to the delta subunit (54). It was further confirmed by the study in which beta clamp mutants deprived of one hydrophobic cleft had a significant alteration in DNA unloading but were efficiently loaded onto the DNA strand (68). In contrast, the DnaN dimer with mutations in both hydrophobic clefts was not loaded onto DNA at all. However, our results indicate that the blockage of hydrophobic clefts *in vivo* may abolish proper interactions of the delta subunit with the beta clamp but may still retain its activity to open the DnaN dimer ring (and, thus, to unload the beta clamp). On the other hand, the catalytic subunit (which binds the tau subunit) may be loaded onto unwound *oriC*, and replication may be initiated (and not restarted in the site of former collapse) even in the absence of the beta clamp. This hypothesis is supported by the observation showing that Alpha-EYFP foci were still observed during GM treatment and that their appearance was followed by duplication of the ParB-mCherry focus, which is attributed to duplication of the *oriC* region. As a result of lost processivity, several chromosomal loops consisting of newly replicated DNA fragments may be generated. We hypothesize that these loops may undergo recombination, which in turn may cause multiplication of *oriC*-proximal regions. In fact, multiplication of the fragment encompassing *oriC* (and the *dnaN* gene) has been reported in GM-resistant strains of M. smegmatis (41). During the GM washout period, we observed multiple DnaN-mCherry foci. We speculate that these DnaN-mCherry foci may be involved in the aforementioned recombination between the *oriC*-proximal loops.

All three tested antibiotics also impacted the overall nucleoid structure. Novobiocin displayed the most modest effect. The bead-like structure of the chromosome was maintained throughout novobiocin treatment, although there was an increase in the distance between individual nucleoid clusters. This stretching of the area occupied by the nucleoid was probably a consequence of the increased cell volume of filamentous cells. GM treatment triggered a more pronounced decondensation of the nucleoid: the bead-like pattern was lost, and there was a decrease in the fluorescence intensity of HupB-EGFP. Loss of the bead-like structure might reflect either overrelaxation of the chromosome or its fragmentation and subsequent cell death (similar to that observed in quinolone-treated E. coli cells [57]). Because the genes encoding both subunits of DNA gyrase are located very near *oriC* in M. smegmatis (approximately 5 kb away from the *oriC* region), the generation of *oriC*-proximal loops might result in multiplication of the *gyrA* and *gyrB* genes and the consequential increased level of gyrase. This might, in turn, affect global supercoiling and overall chromosome organization. This hypothesis is supported by our observation that the Ndx-mediated blockade of gyrase activity led to chromosome compaction. Temporal loss of the regular chromosome structure was also observed in a fraction of GM-treated cells during the washout period, mirroring the heterogeneity that arose during the adaptation to new growth conditions (i.e., the resumption of growth after antibiotic treatment). Interestingly, in some cells exposed to GM, the HupB-EGFP signal was reduced to a single fluorescent spot that became visible for a short time and that then disappeared. This was accompanied by a sudden growth arrest that was not reversed during the washout period. In such cells, only dispersed fluorescence was visible. Similar changes were described earlier in E. coli cells exposed to quinolones (57, 69). It is possible that the frequent collapse of replisomes may, at least in some cells, trigger the SOS response and that the dramatic decrease in fluorescence intensity may be a hallmark of chromosome fragmentation.

Unexpectedly, we did not observe similar changes in Ndx-exposed M. smegmatis cells, and the observed chromosome condensation was reversed upon washout in these cells. This phenomenon, which was accompanied by growth arrest, was strikingly different from that previously reported in E. coli (57, 58). The filamentation of E. coli cells was attributed to initiation of the SOS response after the induction of double-strand breaks by Ndx (70, 71). In the case of M. smegmatis, in contrast, we did not observe similar changes in Ndx-exposed cells. However, our observations are consistent with previous findings that classical quinolones (including Ndx) do not induce the cleavage activity of M. tuberculosis gyrase (72, 73), which is presumably due to the presence of alanine in the 90th position of the GyrA sequence. This residue corresponds to the conserved Ser83 found in many other bacterial species, which has been implicated (together with an acidic residue four positions downstream) in the ability of GyrA to interact with quinolones via a water-metal ion bridge. The replacement of serine with alanine in the corresponding region of M. tuberculosis GyrA is believed to be a key factor responsible for the intrinsic resistance of mycobacteria to quinolones (at least the classical ones).

Interestingly, we observed the extremely different behavior of mycobacterial replisomes and the chromosome as a consequence of inhibiting the same molecular target (i.e., gyrase). The outcome of such inhibition was the same in the case of both novobiocin and Ndx and involved the accumulation of supercoils ahead of replication forks. It is unclear how replisomes were maintained within the replication forks in the case of novobiocin and were disassembled in the case of Ndx. The chromosome decondensed during novobiocin treatment but shrank rapidly during addition of Ndx. The compaction of the nucleoid is also peculiar, in light of a recent study that showed that decondensation of the mycobacterial chromosome is a common response to a broad spectrum of antimicrobials (74). This confirms that the response to different antibiotics may not be uniform even between the same species and that single-cell studies may give invaluable data concerning various agents.

In this work, the between-cell differences in replisome and chromosome dynamics, which were more pronounced at lower inhibitor concentrations (i.e., 5× IC_50_ and 10× IC_50_), likely reflected between-cell differences in cell cycle progression. Future studies measuring individual responses in synchronized bacterial cultures are needed to provide more details about this cell heterogeneity.

Our present work may also prove beneficial in assessing the usefulness of newly developed drugs. Moreover, given that the standard treatment for TB consists of a combination of rifampin, isoniazid, pyrazinamide, and ethambutol for multiple months (75, 76), the system introduced herein may be useful in efforts to determine the optimal drug combination for use against mycobacterial infections and to test new drug combinations. Our results suggest that in mycobacteria, use of a combination of a cell wall synthesis inhibitor with nalidixic acid (or other quinolones) would not likely show any additional benefit beyond the use of Ndx alone, as the cells stopped growing immediately after Ndx was introduced into the medium. On the other hand, it seems that β-lactams may be useful when combined with griselimycin or novobiocin.

## MATERIALS AND METHODS

### Bacterial strains and culture conditions.

Allelic replacement of the genes encoding the alpha (*Msmeg_3178*) or beta (*dnaN*, *Msmeg_0001*) subunits of DNA polymerase III with the *alpha-eyfp* or *dnaN-mCherry* fusion genes, as well as replacement of the *parB* gene with the *parB-mNeon green* or *parB-mCherry* fusion genes, was performed as previously described (47, 48). Replacement of the *hupB* gene with *hupB-egfp* was performed as described by Hołówka and coworkers (49, 50). The mycobacterial strains were cultured in 37°C in 7H9 broth or on 7H10 agar (Difco) supplemented with oleic acid-albumin-dextrose-catalase (OADC; BD), 0.05% Tween 80, and (when needed) proper additives (kanamycin at 50 μg/ml, X-Gal [5-bromo-4-chloro-3-indolyl-β-d-galactopyranoside], 2% sucrose). Correct allelic replacement and proper incorporation of the integration vectors were confirmed using PCR and Western blotting. Fusion of the functional fluorescent protein was confirmed by seminative SDS-PAGE and was visualized using a Typhoon phosphorimager. Western blotting was performed using standard procedures (77) with polyclonal anti-mCherry, monoclonal anti-GFP (Santa Cruz Biotechnology), and monoclonal anti-mNeon (Chromotec) antibodies.

### Determination of IC_50_.

For growth curve analysis, cells were cultured in a Bioscreen C instrument, which is an automated growth curve analyzer. The experiment was performed in a total volume of 300 μl per well, according to the manufacturer’s recommendations, with or without the tested inhibitors (concentration ranges, 0.1 to 1 μg/ml for GM, 30 to 150 μg/ml for Ndx, and 0.5 to 9 μg/ml for novobiocin). Growing conditions were chosen from the manufacturer’s available software options: 37°C, high-speed shaking, normal amplitude, and 3 days. Data were collected every 20 min using a brown filter (600 nm). The growth rate (optical density at 600 nm per minute) was estimated by analyzing the slope of the linearly fitted correlation in the exponential growth phase. The percentage of growth inhibition was calculated by comparing the growth rates obtained in the presence of the tested antibiotics to the growth rate obtained without any inhibitor (which was defined as 100%), as described previously (78). The IC_50_ was calculated for each strain from the inhibition curve plotted using the R package software and was taken as the concentration of a particular compound that inhibited the cell growth rate by 50%.

### TLMM.

TLMM was performed as previously described (47, 78) using B04A plates with an Onix flow-control system (Merck-Millipore). Cells loaded into the observation chamber were exposed to fresh 7H9-OADC-Tween 80 for 5 h, 7H9-OADC-Tween 80-inhibitor for 5 h, and fresh 7H9-OADC-Tween 80 without antibiotic for 7 h. All experiments were performed under continuous pressure (1.5 lb/in^2^) at 37°C. Images were recorded at 2- or 10-min intervals using a Delta Vision Elite inverted microscope equipped with a 100× or 60× oil immersion objective. The exposure conditions were as follows: for the EYFP filter (excitation wavelength, 513/17 nm; emission wavelength, 548/22 nm), 150 ms and 100% intensity; for mCherry (excitation wavelength, 575/25; emission wavelength, 625/45 nm), 80 ms and 50% intensity; and for DIC, 50 ms and 5% intensity. All measurements were taken manually and analyzed with the ImageJ Fiji suite and R software platforms (79).

## Supplementary Material

Supplemental file 1

Supplemental file 2

Supplemental file 3

Supplemental file 4

Supplemental file 5
