# Supplementary material for: Watching DNA Replication Inhibitors in Action: Exploiting Time-Lapse Microfluidic Microscopy as a Tool for Target-Drug Interaction Studies in Mycobacterium
Source: Antimicrob Agents Chemother. 2019 Sep 23;63(10):e00739-19. doi: 10.1128/AAC.00739-19 (PMC6761567; doi:10.1128/AAC.00739-19)
Supplement: Supplemental file 1 [file AAC.00739-19-s0001.pdf]

Supplemental movie legends

Supplemental movie 1. Replication and segregation dynamics during novobiocin treatment. TLMM movies of representative cells of the Alpha-EYFP/DnaN-mCherry (top panel) and the DnaN-mCherry/ParB-mNeon (bottom panel) strains. After 5 hours (300 min) of growth under optimal conditions, the novobiocin ($10xIC_{50}$) was added (+Novo) and then removed 5 hours later (600 min). Images were acquired every 10-min. Scale bar 5 μm.

Supplemental movie 2. Replication and segregation dynamics during Ndx treatment. TLMM movies of representative cells of the Alpha-EYFP/DnaN-mCherry (top panel) and the DnaN-mCherry/ParB-mNeon (bottom panel) strains. After 5 hours (300 min) of growth under optimal conditions, the Ndx ($10xIC_{50}$) was added (+Ndx) and then removed 5 hours later (600 min). Images were acquired every 10-min. Scale bar 5 μm.

Supplemental movie 3. Replication and segregation dynamics during GM treatment. TLMM movies of representative cells of the Alpha-EYFP/DnaN-mCherry (left panel) and the DnaN-mCherry/ParB-mNeon (right top panel) and the Alpha-EYFP/ParB-mCherry (right bottom panel) strains. After 5 hours (300 min) of growth under optimal conditions, the GM ($10xIC_{50}$) was added (+GM) and then removed 5 hours later (600 min). Images were acquired every 10-min. Scale bar 5 μm.

Supplemental movie 4. Chromosome and replisome dynamics during exposure to replication-affecting drugs. TLMM movies of representative cells of the HupB-EGFP/DnaN-mCherry strain during Ndx (left panel), novobiocin (middle panel) and GM (right panel) treatment. After 5 hours (300 min) of growth under optimal conditions, the antibiotics ($10xIC_{50}$) were added (+Ndx, +Novo,

+GM) and then removed 5 hours later (600 min). Images were acquired every 10-min. Scale bar 5 μm.

Supplemental Tables

Strain name	Relevant genotype	Reference
Alpha-EYFP/DnaN-mCherry	*M. smegmatis Mc^2 155 alpha-eyfp, dnaN-mCherry*	(1)
DnaN- mCherry/ParB-mNeon	*M. smegmatis Mc^2 155 dnaN-mcherry , parB-mNeon,*	(2)
Alpha-EYFP/ParB-mCherry	*M. smegmatis Mc^2 155 alpha-eyfp, parB-mCherry*	(1)
DnaN-mCherry/HupB-EGFP	*M. smegmatis Mc^2 dnaN-mcherry, hupB-egfp*	(3)
RRL-19B	*E. coli AB1157 (thr-1, araC14, leuB6(Am), Δ(gpt-proA)62, lacY1, tsx-33, qsr'-0, glnV44(AS), galK2(Oc), LAM-, Rac-0, hisG4(Oc), rfbC1, mgl-51, rpoS396(Am), rpsL31(strR), kdgK51, xylA5, mtl-1, argE3(Oc), thi-1) ypet-dnaN*	(4)

Table S1. Characteristics of strains used in this study

Supplemental Figures

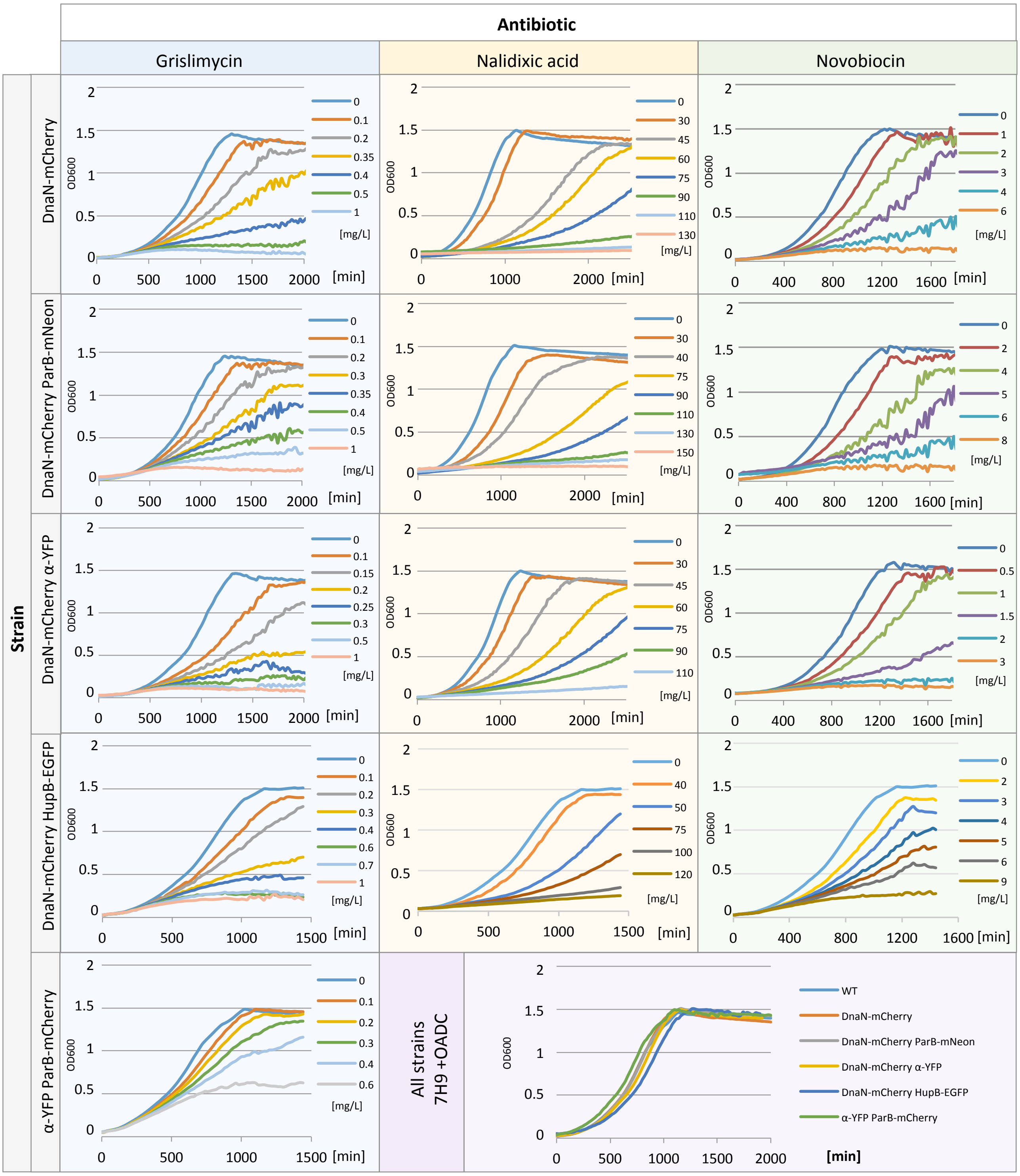

Fig. S1. Growth curves of the *M. smegmatis* strains used in this study in the presence of the different antibiotics. The right bottom panel presents the growth of all strains in antibiotic-free medium.

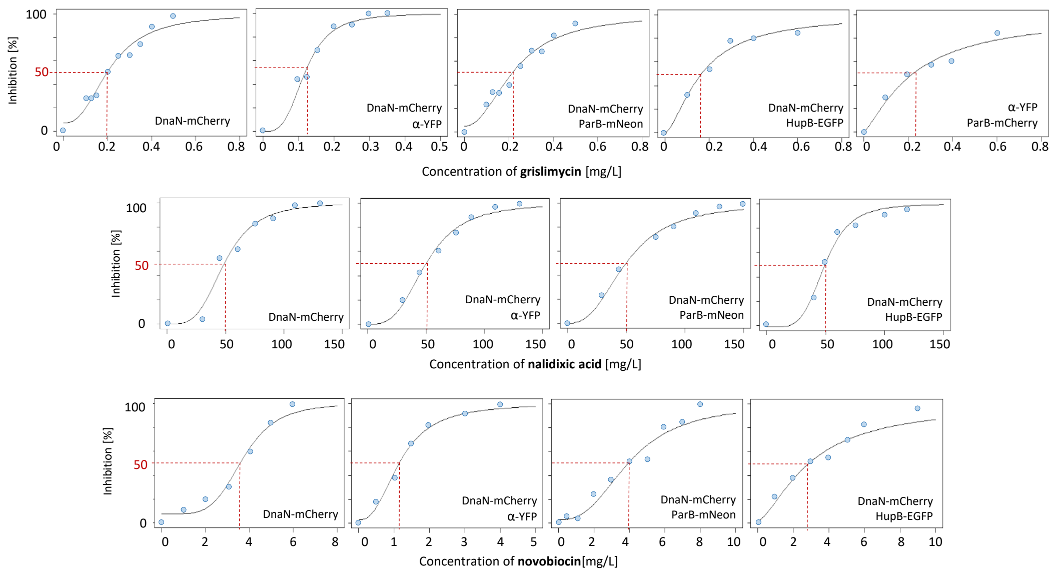

Fig. S2. Inhibition curves calculated from the growth curves plotted in Figure S1. Top panel, griselimycin; middle panel, nalidixic acid; bottom panel, novobiocin. IC_{50} is marked with a red dotted line.

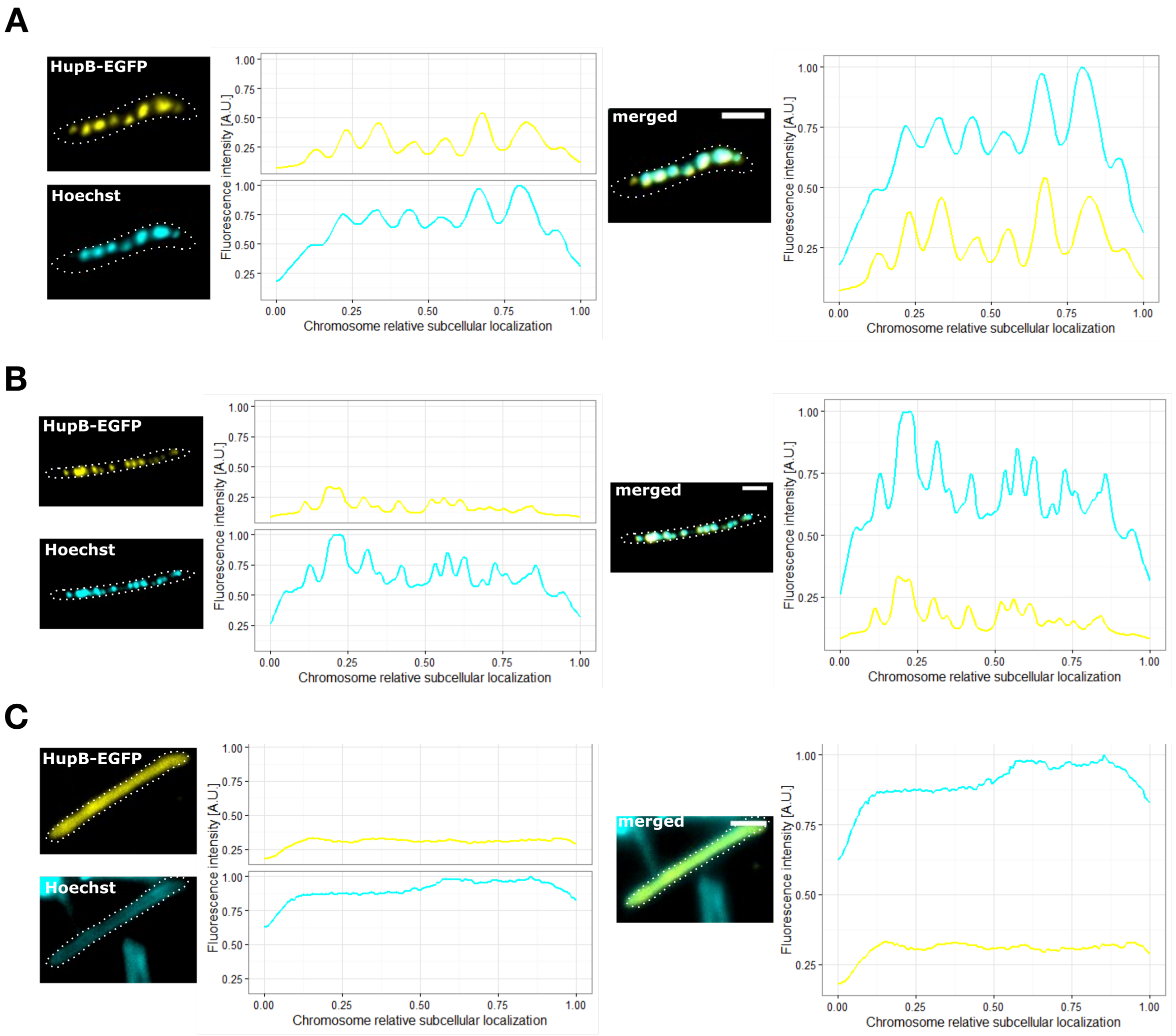

Fig. S3. The binding of HupB-EGFP to the chromosome is not affected under GM treatment, and reflects the nucleoid structure. Micrographs and corresponding fluorescence intensity profiles of DnaN-mCherry/HupB-EGFP cells stained with Hoechst 33342 before (A) and after 5 hours of GM treatment (B and C). During GM treatment, the chromosome is decondensed in some cells (B), while diffuse fluorescence is observed in others (C). Scale bar, 2 μm.

Supplemental references

1. Trojanowski D, Hołówka J, Ginda K, Jakimowicz D, Zakrzewska-Czerwińska J. 2017. Multifork chromosome replication in slow-growing bacteria. Sci Rep 7:43836.

2. Trojanowski D, Ginda K, Pióro M, Hołówka J, Skut P, Jakimowicz D, Zakrzewska-Czerwińska J. 2015. Choreography of the Mycobacterium Replication Machinery during the Cell Cycle. mBio 6:e02125-14.

3. Hołówka J, Trojanowski D, Janczak M, Jakimowicz D, Zakrzewska-Czerwińska J. 2018. The Origin of Chromosomal Replication Is Asymmetrically Positioned on the Mycobacterial Nucleoid, and the Timing of Its Firing Depends on HupB. J Bacteriol 200(10) e00044-18.

4. Reyes-Lamothe R, Sherratt DJ, Leake MC. 2010. Stoichiometry and Architecture of Active DNA Replication Machinery in Escherichia coli. Science 328:498–501.
